# Idiopathic Multicentric Castleman Disease With Severe Eosinophilia and Diffuse Centrilobular Nodule—A Rare Case Report

**DOI:** 10.1155/crh/7761718

**Published:** 2025-12-11

**Authors:** Xiaojuan Li, Shuang Li, Tianming Zhao, Biao Xu, Xinge Du, Xinyu Song, Yingnan Wang

**Affiliations:** ^1^ Department of Respiratory and Critical Care Medicine, Yichang Central People’s Hospital, The First College of Clinical Medical Science, China Three Gorges University, Yichang, Hubei, China, ctgu.edu.cn; ^2^ Department of Pathology, Yichang Central People’s Hospital, The First College of Clinical Medical Science, China Three Gorges University, Yichang, Hubei, China, ctgu.edu.cn; ^3^ Third-Grade Pharmacology Laboratory of Traditional Chinese Medicine, State Administration of Traditional Chinese Medicine, China Three Gorges University, Yichang, Hubei, China, ctgu.edu.cn

**Keywords:** case report, Castleman disease, centrilobular nodules, eosinophilia

## Abstract

**Rationale:**

Idiopathic multicentric Castleman disease (iMCD), also known as angiofollicular lymph node hyperplasia, is a rare inflammatory lymphoproliferative disease with diverse clinical presentations. We report a rare case of iMCD accompanied by severe eosinophilia and diffuse centrilobular pulmonary nodules, which have rarely been previously documented in the literature.

**Patients’ Concerns:**

A 69‐year‐old man presented with intermittent fever, dry cough, and shortness of breath. Laboratory examination revealed severe eosinophilia. Chest computed tomography (CT) revealed bilateral pulmonary interstitial nodules and enlarged lymph nodes in the right axilla and mediastinum.

**Diagnosis:**

Axillary lymph node biopsy revealed partial atrophy of lymphoid follicles with hyaline vessel insertion and partial hyperplasia. The hyperplastic mantle zones were composed of concentric rings of small lymphoid cells. Additionally, numerous plasma cells and eosinophils were observed infiltrating between the follicles. The patient was ultimately diagnosed with iMCD with eosinophilia. Other potential causes of eosinophilia, including infections, malignancies, and other inflammatory conditions, were excluded.

**Intervention:**

The patient declined cytotoxic chemotherapy and was treated with oral methylprednisolone (40 mg/day), which was gradually tapered to 10 mg/day.

**Outcomes:**

The patient’s symptoms, including fever, cough, and dyspnea, improved markedly. The eosinophil count returned to normal, and inflammatory cytokine levels (IL‐1β, IL‐8, IL‐6, and TNF‐α) decreased significantly.

**Lessons:**

This case highlights a rare presentation of iMCD with eosinophilia and pulmonary involvement, emphasizing the importance of early recognition and timely corticosteroid therapy. Our report adds to the limited data on iMCD with eosinophilia and may help inform future clinical management.

## 1. Introduction

Castleman disease is a rare lymphoproliferative disorder first described by Benjamin Castleman in 1956, with unclear etiology and pathogenesis [1]. Based on the distribution of the enlarged lymph nodes, Castleman disease is clinically categorized into unicentric Castleman disease (UCD) and multicentric Castleman disease (MCD). MCD can be categorized based on the presence or absence of human herpes virus 8 (HHV8) into two subtypes: HHV8‐associated MCD and idiopathic MCD (iMCD) [2, 3]. The latter can present with a variety of clinical symptoms, such as fever, lymphadenopathy, and organomegaly. iMCD has been further categorized into several subtypes based on clinical and pathological features, such as TAFRO syndrome, among which iMCD with eosinophilia is a particularly rare manifestation [4].

Although several cases of iMCD with eosinophilia have been reported, they typically lack pulmonary involvement or only present with mild respiratory symptoms. For instance, Ishii et al. reported a case of MCD with severe eosinophilia, but without notable lung imaging abnormalities [5]. Similarly, Mima et al. described an iMCD case with eosinophilia in the absence of lung findings [6]. Another case by Katsumata et al. involved a 67‐year‐old woman initially misdiagnosed with eosinophilic pneumonia; she exhibited prominent eosinophil and IgG4‐positive plasma cell infiltration in both lung and lymph node tissue. However, the pulmonary imaging mainly showed interstitial changes rather than diffuse centrilobular nodules [7]. In a larger cohort study, Zhou et al. reported that approximately 36% of patients with iMCD‐NOS had pulmonary involvement, most commonly manifesting as nodules, cysts, or areas of consolidation. However, the study did not specifically address the presence of eosinophilia or describe centrilobular nodule patterns [8].

In contrast, our patient presented with persistent and marked eosinophilia along with bilateral diffuse centrilobular nodules on CT, a combination that, to our knowledge, has not been previously reported.

This case adds valuable clinical insight into the rare eosinophilic variant of iMCD, highlighting the potential role of cytokines such as IL‐6 in pathogenesis, and underscores the importance of early recognition and individualized treatment. Recent studies support the use of corticosteroid therapy in iMCD, especially in eosinophilic forms [9].

Table 1 summarizes selected published cases and studies on iMCD with eosinophilia and/or pulmonary involvement. Our case appears to be the first to document the coexistence of persistent eosinophilia and diffuse centrilobular nodules in an iMCD patient.

**Table 1:** Summary of selected published cases and studies on iMCD with eosinophilia and/or pulmonary involvement.

Report	Age/gender	Eosinophilia	Lung involvement	IL‐5/IL‐6 data
Ishii et al. [5]	40/M	Severe	No	IL‐5↑
Katsumata et al. [7]	67/M	Prominent	Yes (interstitial involvement)	Not reported
Mima et al. [6]	68/Male	Severe	No	IL‐6↑
Zhou et al. [8]	162 iMCD‐NOS cases	Not described	Yes (36.4% had nodules, cysts, or consolidation)	Not evaluated
This case	69/M	Severe	Yes (diffuse nodules)	IL‐6↑

## 2. Case Presentation

A 69‐year‐old male presented with intermittent fever, dry cough, and shortness of breath for six months, with no significant medical history. On physical examination, an enlarged lymph node was noted in the right axilla, measuring approximately 2 cm in its largest dimension. Decreased bilateral breath sounds with a few wet rales were observed, and chest computed tomography (CT) revealed multiple micronodular infiltrates in the bilateral lung interstitium (Figure 1(a)) and numerous enlarged lymph nodes in the right axilla and mediastinum (Figures 1(b) and 1(c)), which raised concerns for pulmonary involvement in a systemic inflammatory condition.

Figure 1Chest CT image of the patient. (a) Multiple micronodular infiltrates in the bilateral lung interstitium; (b, c) numerous enlarged lymph nodes in right axilla and mediastinum.

**Figure:**
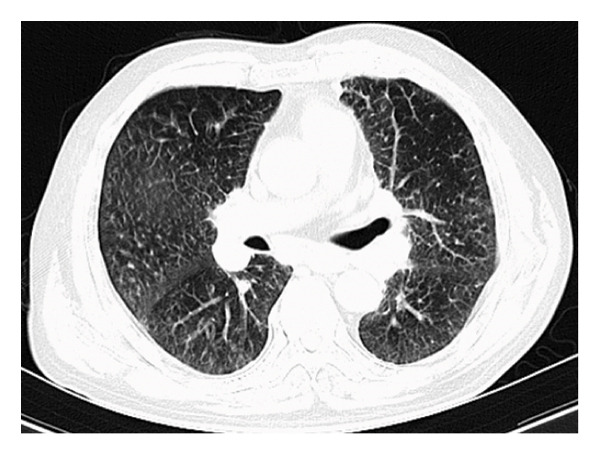
(a)

**Figure:**
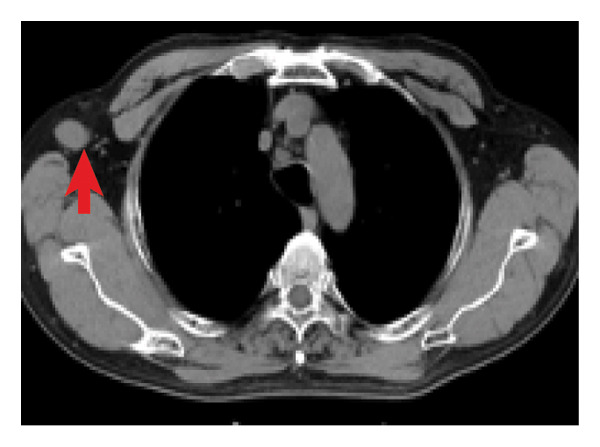
(b)

**Figure:**
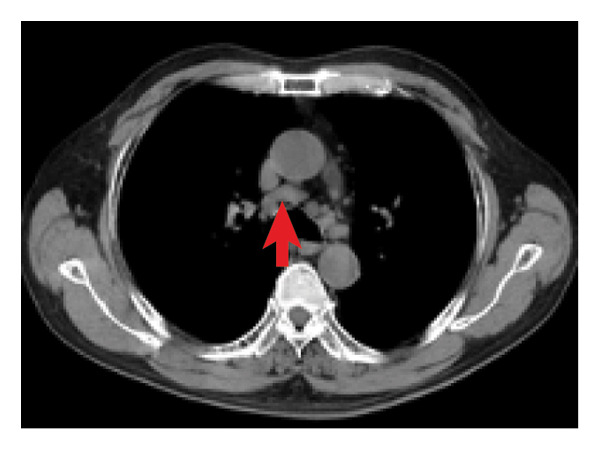
(c)

Laboratory results, as indicated in Table 1, showed an abnormal white blood cell counts, severe eosinophilia, and elevated inflammatory markers, including IL‐6 and tumor necrosis factor‐α (TNF‐α). These findings suggested a possible eosinophilic disorder. A comprehensive differential diagnosis was conducted, including sarcoidosis, IgG4‐related diseases, lymphoma, and eosinophilic granulomatosis with polyangiitis (EGPA).

An axillary lymph node biopsy was performed, revealing lymphoid follicles with partial atrophy and concentric rings of small lymphoid cells, along with plasma cells and eosinophil infiltration, consistent with iMCD. No signs of infection, malignancy, or IgG4‐related disease were found. The patient was ultimately diagnosed with iMCD accompanied by marked eosinophilia, which is a very rare clinical manifestation.

It is characterized by a proliferative mantle region consisting of concentric rings of small lymphoid cells. In addition, it was found that many plasma cells and eosinophils infiltrated between the follicles, without the presence of RS‐like large cells. HHV8 tested negative in lymph nodes (Figures 2(a), 2(b), and 2(c)). Immunohistochemistry showed positive for CD3, CD20, CD21, Ki‐67 (with high expression in the germinal center), CD138, lambda, IgG, CD10 (in the germinal center), BCL‐2 (outside the germinal center), and BCL‐6 (in the germinal center) but negative for IgG4.

Figure 2Pathological images of the patient’s lymph node and bone marrow. (a) Typical “onion skin‐like” pattern, and hyaline degeneration of small blood vessels (HE × 200); (b) diffusely distributed plasma cells and lymphocytes (HE × 200); (c) negative expression for HHV‐8 (HHV‐8 × 200); (d) bone marrow biopsy pictures of the patient (HE × 100).

**Figure:**
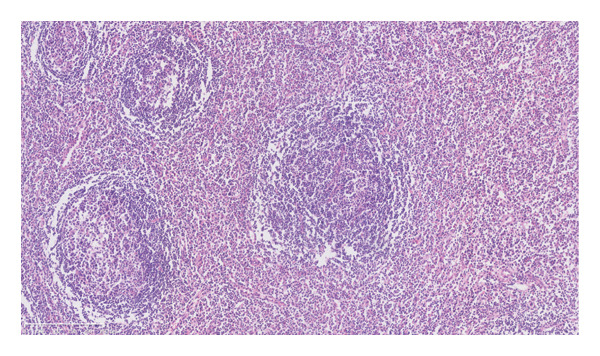
(a)

**Figure:**
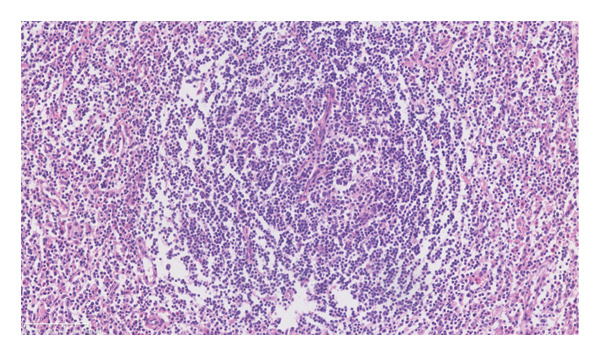
(b)

**Figure:**
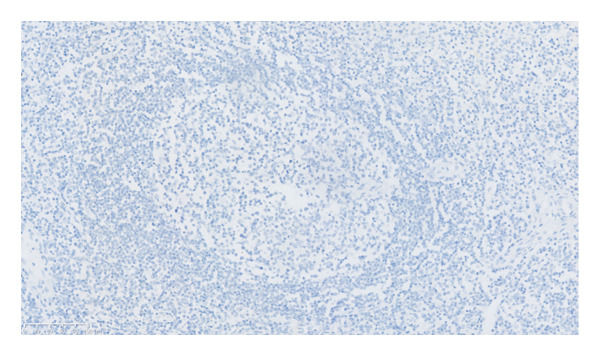
(c)

**Figure:**
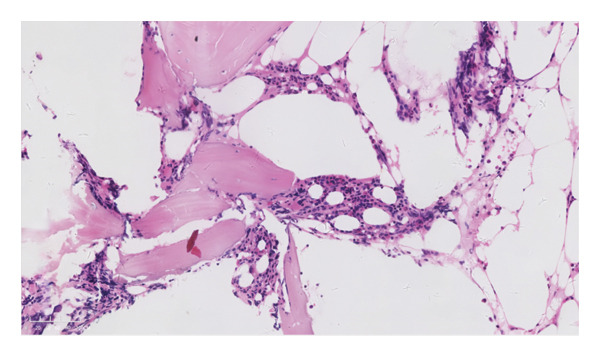
(d)

MCD is often associated with cytokine alterations. In this case, serum levels of IL‐1β, IL‐8, IL‐6, IL‐2 receptor, and TNF‐α were elevated. Notably, eosinophil levels in this patient were abnormally high. Bone marrow cytology revealed myeloproliferation dominated by mature eosinophils, and a bone marrow biopsy indicated myeloid hypoplasia with prominent eosinophils (Figure 2(d)). A FISH assay did not detect rearrangements of CBFβ, FGFR1, JAK2, and PDGFRB; deletion of CHIC2; or FIP1L1/PDGFRA fusion. No abnormal primary cells were found in the bone marrow examination, thus excluding an eosinophilia secondary to leukemic disorders.

The patient was diagnosed with iMCD, accompanied by eosinophilia. Due to the patient’s refusal to use cytotoxic drugs such as cyclophosphamide and vincristine, treatment was commenced with the oral administration of 40 mg of methylprednisolone. This dosage was gradually tapered to 10 mg. During the course of treatment, the patient’s symptoms, including fever, cough, and dyspnea, improved. After approximately 3 years of treatment, a re‐evaluation of the patient’s laboratory indicators revealed that the eosinophil count was normalized. Additionally, cytokines such as IL‐1β, IL‐8, IL‐6, IL‐2 receptor, and TNF‐α had significantly decreased (Table 2), suggesting a positive therapeutic response.

**Table 2:** Parameters of the patient.

Parameters	Before treatment	After treatment	Normal range
WBC (10^9^/L)	11.79	9.24	3.5–9.5
Neutrophils (10^9^/L)	5.5	6.61	1.8–6.3
Eosinophilia (10^9^/L)	3.17	0.20	0.02–0.52
Basophils (10^9^/L)	0.12	0.06	0–0.06
Monocytes (10^9^/L)	0.67	0.58	0.1–0.6
RBC (10^12^/L)	4.62	3.78	4.3–5.8
Hemoglobin (g/L)	114	110	130–175
PLT (10^9^/L)	338	429	125–350
Peripheral blood cell Morphology	Normal	Not assessed	
CD4^+^ T‐cells (%)	48	Not assessed	30–46
CD8^+^T‐cells (%)	19	Not assessed	19.17–33.63
CD4^+^/CD8^+^ ratio	2.46	Not assessed	0.95–2.12
Total protein (g/L)	106.4	55.6	65–85
Albumin (g/L)	37.5	32.3	40–55
Globulin (g/L)	68.9	23.3	20–35
Creatinine (umol/L)	115	95	57–111
Uric acid (umol/L)	599	453	208–428
Immunoglobulin IgG (g/L)	54.77	6.59	7–16
Complement C3 (g/L)	0.59	1.32	0.9–1.8
Complement C4 (g/L)	0.03	0.30	0.1–0.4
ALT (U/L)	40	23.6	9–50
AST (U/L)	35	13.0	15–40
Serum sodium (mmol/L)	140	144.8	135–145
Serum potassium (mmol/L)	3.9	3.81	3.5–5.5
Serum calcium (mmol/L)	2.4	2.23	2.25–2.75
HIV, HBV, and HCV	Negative		
IL‐1β (pg/mL)	60	5.8	0–5
IL‐6 (pg/mL)	112	9	0–7
IL‐8 (pg/mL)	445	42.6	0–62
IL‐2 receptor (U/mL)	2500	1477	233–710
TNF‐α (pg/mL)	31.70	22.3	0–8.1

*Note:* ALT: alanine aminotransferase; AST: aspartate aminotransferase; PLT: platelet; IL‐1β: Interleukin‐1β, IL‐8: Interleukin‐8, IL‐6: Interleukin‐6, IL‐2 receptor: Interleukin‐2 receptor.

Abbreviations: HBV, Hepatitis B virus; HCV, Hepatitis C virus; HIV, human immunodeficiency virus; RBC, red blood cell count; TNF‐α, tumor necrosis factor‐α; WBC, white blood cell count.

## 3. Discussion

This case presents a rare manifestation of iMCD, with significant eosinophilia and pulmonary involvement, contributing to the limited number of documented cases of iMCD with eosinophilia. The patient’s symptoms were similar to those seen in eosinophilic conditions, such as EGPA, though key clinical features of EGPA, such as vasculitis and hematuria [10], were absent.

Eosinophil counts typically change in response to parasitic infections, primarily aiding the host’s defense against these parasites [11, 12]. Notably, IgG levels against common parasites were negative, ruling out a parasitic infection. Eosinophilia can also occur in rheumatoid arthritis, though it is uncommon in early stages [13]. The lack of swelling, pain, or deformity in the patient’s extremities, along with negative results for rheumatoid factors and anticyclic citrullinated peptide antibody, led us to exclude rheumatoid arthritis. Autoantibodies, including ANA, ENA, and ds‐DNA, were also negative, eliminating the possibility of systemic lupus erythematosus (SLE).

Furthermore, FISH tests examining rearrangements of CBFβ, FGFR1, JAK2, and PDGFRB, as well as the deletion of CHIC2 and the FIP1L1/PDGFRA fusion, were conducted. These abnormalities could indicate a hematologic tumor originating from eosinophils, such as chronic eosinophilic leukemia [14]. However, no primary cells were detected in the bone marrow, and no signs of hematological malignancies resulting in elevated eosinophils were found. Additionally, despite the patient’s elevated serum IgG levels, an increase in IgG4 was not observed, making the likelihood of an IgG4‐related disease unlikely. Hypoadrenalism was also ruled out due to normal cortisol levels. Finally, an axillary lymph node biopsy revealed partial atrophy of lymphoid follicles with hyaline vessel insertion and partial hyperplasia, with a negative result for HHV8 in the lymph node. Ultimately, the patient was diagnosed with iMCD.

Although the lymphadenopathy was limited to the right axilla and mediastinum, the presence of systemic symptoms (fever and elevated CRP) and significantly increased inflammatory cytokines (IL‐6 and TNF‐α) fulfilled the international diagnostic criteria for iMCD [2]. These findings support a diagnosis of iMCD disease rather than oligocentric CD.

Eosinophilia in iMCD has been sparsely documented. Elevated eosinophil levels have been linked to systemic inflammatory conditions and may be a response to cytokines such as IL‐5 and IL‐6, which are typically elevated in iMCD [5]. Among these, IL‐6 is particularly significant in the development and progression of Castleman disease. Elevated serum IL‐6 levels are positively correlated with systemic inflammatory symptoms and organ damage [15], which typically decrease following treatment and correspond with the improvement of symptoms. The exact cause of this cytokine level increase remains unclear.

Although IL‐5 was not measured in this case due to institutional limitations, it plays a well‐established role in eosinophil activation and recruitment. Previous reports of iMCD with eosinophilia have demonstrated elevated IL‐5 levels [5], suggesting that IL‐5 may contribute to the pathophysiology in such cases. While IL‐6 blockades (e.g., siltuximab or tocilizumab) remains the mainstay of treatment in iMCD [2, 15], its indirect effects on eosinophil‐regulating cytokines such as IL‐5 may partly explain its efficacy in eosinophilic variants. Further studies are warranted to explore whether dual cytokine‐targeted strategies might benefit this subset of patients.

Chest imaging in cases of MCD often shows lymphocytic interstitial pneumonia. This condition typically presents as a diffuse distribution of spots, nodules, or patch‐like shadows in both lungs, distributed along the bronchial vascular bundle and pleura, with localized interlobular septal thickening [16, 17].

Pulmonary involvement in MCD can manifest as lymphocytic interstitial pneumonia or nodular changes [16]. In this case, diffuse centrilobular nodules observed on CT, combined with severe eosinophilia, raised the question of direct lymphoproliferative infiltration versus a secondary inflammatory response. Due to the lack of histopathological confirmation from lung tissue, we interpret the pulmonary findings as more likely secondary to cytokine‐driven inflammatory processes—such as IL‐6‐ or IL‐5‐mediated eosinophilic inflammation—rather than direct Castleman‐related infiltration. The gradual resolution of pulmonary nodules following corticosteroid therapy further supports this inflammatory mechanism.

The treatment for iMCD with eosinophilia in this case involved corticosteroids, which are commonly used to treat eosinophilic disorders [18–20]. This case underscores the importance of recognizing eosinophilia in patients with iMCD, as it may present a distinct challenge in diagnosis and treatment. The patient’s response to corticosteroids supports the existing literature on the efficacy of steroids in managing eosinophilic disorders. However, the rarity of such presentations highlights the need for further research into optimal treatment strategies for iMCD with eosinophilia.

## 4. Conclusion

This report details an exceptionally rare case of iMCD presenting with severe eosinophilia and bilateral pulmonary nodules. This case report provides valuable insights into this rare manifestation, contributing to the understanding of this complex disease and suggesting potential therapeutic strategies for similar cases.

**Nomenclature**
MCD: Multicentric Castleman disease
iMCD: Idiopathic multicentric Castleman disease
HHV‐8: Human Herpesvirus 8

## Ethics Statement

The present case study was approved by the ethics committee of the People’s Hospital of China Three Gorges University (No. PJ2022‐23). The patient allowed personal data processing. Informed consent was also obtained from the patient.

## Consent

Please see the Ethics Statement.

## Conflicts of Interest

The authors declare no conflicts of interest.

## Author Contributions

Conceptualization: Xiaojuan Li, Xinyu Song, and Xinge Du.

Data curation: Xiaojuan Li, Shuang Li, Xinyu Song, Tianming Zhao, and Biao Xu.

Funding acquisition: Tianming Zhao and Xiaojuan Li.

Writing–review and editing: Xiaojuan Li, Shuang Li, Tianming Zhao, and Yingnan Wang.

## Funding

This work has been supported by the Natural Science Foundation of Medical and Health Research Project of Yichang (A23‐1‐025, A24‐2‐026).
